# A Learner-Driven Workshop to Enhance Feedback Engagement in Emergency Medicine

**DOI:** 10.7759/cureus.103910

**Published:** 2026-02-19

**Authors:** Allison M Beaulieu, Brian Merritt, Julia Ruggieri, Rowan H Kelner, Christine Raps, Patrick Hughes, Jeffrey Druck, Megan Fix, Robert Stephen

**Affiliations:** 1 Emergency Medicine, The University of Utah, Salt Lake City, USA

**Keywords:** emergency medicine, feedback, learner-driven, medical education, resident education

## Abstract

Introduction: Feedback is fundamental to Emergency Medicine (EM) education; however, residents frequently encounter obstacles when attempting to obtain and implement it. Learner-driven feedback strategies may strengthen feedback culture; however, methods to develop feedback literacy, the ability of learners to understand, value, and effectively use feedback, remain largely underexplored. We developed and evaluated a workshop to prepare EM residents to actively engage in the feedback process.

Methods: A prospective pre-post survey was conducted at a single academic EM residency. Postgraduate year (PGY) 1-3 residents participated in a 1.5-hour interactive, practice-based workshop that included didactic components and hands-on activities focused on clarifying expectations, goal setting, and receiving feedback. Residents completed pre- and post-surveys using a five-point Likert scale to assess comfort. Knowledge retention was assessed one week later with a 15-question assessment. Pre- and post-surveys were evaluated by paired t-test analysis.

Results: Thirty-one residents completed the pre-survey, and 30 completed both the post-survey and the knowledge assessment. Statistically significant improvements were observed in resident comfort for clarifying expectations (Δ = 0.67; p < 0.001), creating goals (Δ = 0.90; p < 0.001), comfort receiving feedback (Δ = 0.33; p = 0.01), seeking feedback (Δ = 0.40; p = 0.02), creating feedback action plans (Δ = 1.70; p < 0.001), reflecting on and implementing feedback (Δ = 0.40; p < 0.001), and recognizing feedback as the learner’s responsibility (Δ = 0.53; p = 0.002). Perceptions of feedback’s importance and impact on patient care remained high and unchanged. Knowledge retention among residents was high, with 91.1% of items (247/270) answered correctly. The strongest performance was observed in the domains of Expectations (87/90, 96.7%) and Goal Setting (88/90, 97.8%), while the Feedback Action Plan domain showed the lowest scores (73/90, 81.1%).

Conclusion: A structured workshop significantly improved EM residents’ comfort, knowledge, and skills in engaging with feedback. Early introduction of learner‑driven strategies may strengthen feedback culture and support professional development. This learner‑centered model represents a meaningful shift from traditional faculty‑directed feedback frameworks by placing primary ownership of the process in the hands of learners. Further research is needed to assess long‑term retention, clinical application, and the role of faculty development.

## Introduction

Emergency Medicine (EM) residency training encompasses three or four years of rigorous clinical practice, structured didactics, and significant professional development. The Accreditation Council for Graduate Medical Education (ACGME) program requirements for EM residency mandate that faculty provide residents with frequent feedback and that residents demonstrate the ability to incorporate this feedback into their performance [1]. Effective feedback delivery has been a frequent topic of research in medical education and other fields [2-4]. In just a few years, residents must rapidly advance their knowledge and skills to prepare for board certification and independent practice. When given effectively, feedback is a valuable tool that allows learners to focus their attention where it is most needed [5]. Best practices suggest encouraging learners to participate in the feedback process actively [3]. In competency-based medical education (CBME), feedback is essential for guiding progression toward milestones and entrustable professional activities, serving as the link between assessment and learning [3]. Despite this, limited research exists on strategies to promote resident engagement in seeking and receiving feedback. The investigation into the utility of a learner-centric feedback structure remains an area for continued growth, particularly in the field of EM. 

The term “feedback culture” describes an environment where feedback is welcomed, solicited, and received frequently [3]. However, establishing such a culture can be challenging. The dynamics of shift work and the environment of the emergency department often make giving feedback difficult [6]. There are also factors of psychological safety, with many residents reporting stress in association with receiving feedback and very few citing a positive experience [7]. In spite of these challenges, residents report wanting to receive constructive criticism from faculty [8]. Moreover, the quality and quantity of feedback improve in environments where learner-driven feedback is utilized [9]. Shifting to a learner-driven feedback model may promote improvements in feedback culture [9]. 

Despite widespread emphasis on feedback in graduate medical education, most interventions focus on improving faculty delivery rather than empowering learners to actively shape the process. This gap limits the development of feedback literacy, a critical skill for lifelong learning and professional growth. To address this need, we designed an innovative workshop that shifts the paradigm from passive receipt to active engagement, equipping EM residents with practical strategies to solicit and apply high-quality feedback. This workshop creates an opportunity to investigate the impact of a learner-centric feedback curriculum on emergency medicine residents. 

Unlike traditional approaches, this learner-centered intervention integrates four actionable components: clarifying expectations, setting Specific, Measurable, Achievable, Relevant, and Time-Bound (SMART) goals, creating a personalized feedback action plan, and adopting a growth mindset when receiving feedback. By providing a structured framework for proactive feedback behaviors, this workshop aims to cultivate a sustainable feedback culture and enhance residents’ ability to leverage feedback throughout training and beyond.

## Materials and methods

Study design

This prospective, pre-post survey-based study was conducted at a single EM residency program in the United States. The intervention, a 1.5-hour interactive, practice-based workshop, was designed to equip residents with skills to actively enhance the quality and quantity of feedback they receive. The premise for this program was grounded in evidence-based principles of feedback literacy and learner-driven feedback strategies, which emphasize empowering learners to clarify expectations, set goals, and engage meaningfully in the feedback process.

Postgraduate years (PGY) 1-3 residents participated in the workshop, which combined didactic instruction with hands-on activities focused on three core areas: clarifying expectations, creating SMART goals, and receiving feedback with a growth mindset. The workshop was developed and delivered by two physicians with master’s-level training in medical education, ensuring alignment with best practices in adult learning and competency-based education.

Study setting and population

The population evaluated in this study was EM residents (PGY1-3) at a single, academic residency program. The study was conducted during the program’s transition to the new academic year, when PGY-1 to PGY-3 residents were present. 

Study protocol

Prior to the workshop, learners completed a pre-survey assessing their comfort and knowledge regarding the core content areas (Appendix 1). Immediately following the workshop, the same survey was administered to measure immediate changes in perceived comfort and understanding. This approach allowed for direct comparison of pre- and post-intervention responses using a consistent instrument. To evaluate short-term retention of material, a knowledge assessment was conducted one week after the workshop. This consisted of nine multiple-choice questions (Appendix 2) covering key concepts from the session. This component allowed for the investigation of retention of knowledge from the workshop, as well as adding a knowledge-based assessment to the study, in addition to the self-reported survey responses. 

Measurements

The surveys were scored on a five-point Likert scale (1-Strongly Disagree to 5-Strongly Agree). The assessment questions were graded based on correct and incorrect responses, as written by the lecturers. 

Data analysis

Survey and quiz data were analyzed using R version 4.3.1 (R Core Team, R Foundation for Statistical Computing, Vienna, Austria) [10]. Paired pre- and post-survey responses were compared using two-tailed paired t-tests for each item. Mean scores and standard deviations were reported, with statistical significance set at p < 0.05. Quiz performance was summarized using the percentage of correct answers for each item and grouped into three content domains: expectations, SMART goals, and feedback plan. Descriptive statistics were stratified by PGY level.

## Results

The residency program has a total of 36 residents. All 31 residents in attendance for the session completed the pre-survey, while 30 residents completed both the pre- and post-session surveys, and 30 residents completed the post-session assessment. Twenty-eight residents completed both surveys and the post-session evaluation. Of the 31 who completed the pre-survey, 19 (61.3%) identified as female and 12 (38.7%) as male. Participants represented a range of postgraduate training levels: 11 (35.5%) were PGY-1 residents, 11 (35.5%) were PGY-2 residents, and nine (29.0%) were PGY-3 residents (Table 1).

**Table 1:** Participant demographics (pre-workshop survey, N = 31)

Category	Count	Percentage
Gender		
Female	19	61.3%
Male	12	38.7%
Postgraduate year (PGY)		
PGY-1	11	35.5%
PGY-2	11	35.5%
PGY-3	9	29.0%

Thirty residents completed pre- and post-session surveys (Table 2). Statistically significant improvements were observed across multiple domains, including clarifying expectations (Δ = 0.67; p < 0.001), creating SMART goals (Δ = 0.90; p < 0.001), comfort receiving feedback (Δ = 0.33; p = 0.01), seeking feedback (Δ = 0.40; p = 0.02), creating feedback action plans (Δ = 1.70; p < 0.001), reflecting on and implementing feedback (Δ = 0.40; p < 0.001), and recognizing feedback as the learner’s responsibility (Δ = 0.53; p = 0.002). No significant change was observed in the perception that residents take feedback personally (Δ = 0.23; p = 0.24), feedback is important for growth (Δ = 0.2; p = 0.056), or in beliefs regarding the impact of feedback on patient care (Δ = 0.13; p = 0.10). 

**Table 2:** Pre- and post-workshop survey scores by item (N = 30, 1-Strongly Disagree to 5-Strongly Agree) * meets statistical significance with p-value < 0.05 SMART: Specific, Measurable, Achievable, Relevant, and Time-Bound

Survey Item	Pre Mean (SD)	Post Mean (SD)	Δ (Post − Pre)	t	p-value
Clarify expectations from feedback	3.87 (0.68)	4.53 (0.51)	+0.67	-5.53	< 0.001 *
Define SMART goals	3.80 (0.71)	4.70 (0.47)	+0.90	-6.92	< 0.001 *
Open to receiving constructive feedback	3.03 (1.16)	3.27 (1.36)	+0.23	-1.19	0.243
Comfortable asking for feedback	4.00 (0.64)	4.33 (0.55)	+0.33	-2.76	0.010 *
Proactively seeks feedback	3.77 (0.94)	4.17 (0.59)	+0.40	-2.56	0.016 *
Uses a feedback plan	2.83 (0.87)	4.53 (0.57)	+1.70	-9.43	< 0.001 *
Reflects and applies feedback	4.13 (0.57)	4.53 (0.51)	+0.40	-3.89	< 0.001 *
Feedback supports professional growth	4.60 (0.56)	4.80 (0.41)	+0.20	-1.99	0.056
Feedback improves patient care	4.67 (0.48)	4.80 (0.41)	+0.13	-1.68	0.103
Learners are responsible for feedback	3.77 (0.82)	4.30 (0.65)	+0.53	-3.40	0.002 *

Thirty residents completed the post-workshop knowledge quiz, which consisted of nine multiple-choice questions covering key concepts from the session. Overall, residents performed well, answering 247 out of 270 items correctly (91.1%) across all training levels. PGY-1 residents scored 85/90 (94.4%), PGY-2 residents 102/108 (92.6%), and PGY-3 residents 62/72 (86.1%) (Table 3). Most residents answered the majority of questions correctly. The item with the lowest correct response rate concerned the ideal timing for initiating feedback discussions during a four-week rotation, with only 5/10 (50.0%) PGY-1 residents, 5/12 (41.7%) PGY-2 residents, and 5/8 (62.5%) PGY-3 residents selecting the correct answer (Table 3). Performance was highest in the Expectations (87/90, 96.7%) and Goal Setting (88/90, 97.8%) domains, with slightly lower scores in the Feedback Action Plan domain (73/90, 81.1%). 

**Table 3:** Knowledge assessment by question item accuracy and percentage correct by sub-category and postgraduate level PGY: postgraduate year; SMART: Specific, Measurable, Achievable, Relevant, and Time-Bound

Question Item	PGY-1 (n=10)	PGY-2 (n=12)	PGY-3 (n=8)	All Residents (n=30)
Expectations				
Purpose of feedback	10/10 (100.0%)	12/12 (100.0%)	7/8 (87.5%)	29/30 (96.7%)
When to ask about expectations	10/10 (100.0%)	12/12 (100.0%)	7/8 (87.5%)	29/30 (96.7%)
Resident's role in feedback	10/10 (100.0%)	12/12 (100.0%)	7/8 (87.5%)	29/30 (96.7%)
Expectation Totals	30/30 (100.0%)	36/36 (100.0%)	21/24 (87.5%)	87/90 (96.7%)
Goal Setting				
S in SMART	10/10 (100.0%)	11/12 (91.7%)	7/8 (87.5%)	28/30 (93.3%)
SMART goals benefit	10/10 (100.0%)	12/12 (100.0%)	7/8 (87.5%)	29/30 (96.7%)
Best goal-setting practice	10/10 (100.0%)	12/12 (100.0%)	8/8 (100.0%)	30/30 (100.0%)
Goal Setting Totals	30/30 (100.0%)	36/36 (100.0%)	22/24 (91.7%)	88/90 (97.8%)
Feedback Action Plan				
Mid-point check-in benefit	10/10 (100.0%)	12/12 (100.0%)	7/8 (87.5%)	29/30 (96.7%)
Benefit of the feedback plan	10/10 (100.0%)	12/12 (100.0%)	7/8 (87.5%)	29/30 (96.7%)
Ideal time for feedback	5/10 (50.0%)	5/12 (41.7%)	5/8 (62.5%)	15/30 (50.0%)
Feedback Action Plan Totals	25/30 (83.3%)	29/36 (80.6%)	19/24 (79.2%)	73/90 (81.1)
Overall Assessment	85/90 (94.4%)	102/108 (92.6%)	62/72 (86.1%)	247/270 (91.0%)

## Discussion

Feedback is widely recognized as a cornerstone of clinical medical education, yet much of the literature focuses on faculty-driven delivery rather than learner-driven engagement [11,12]. While feedback-seeking behavior is known to correlate with improved performance and professional growth, residents often lack the tools, confidence, and institutional culture to initiate and structure feedback effectively [13-17]. This workshop addresses that gap by introducing a learner-centered framework that empowers residents to clarify expectations, set SMART goals, and co-create actionable feedback plans while maintaining a growth mindset. Grounded in educational theory and designed for EM trainees, the intervention represents a novel approach to improving feedback literacy early in residency and providing residents a framework to lead a learner-centric feedback model [16-18].

The workshop led to significant improvements in residents’ comfort with both receiving and seeking feedback, as well as their ability to reflect on and implement it. Notably, there was a dramatic increase in the use of structured feedback plans, suggesting that residents felt more equipped to engage proactively with faculty. The pre-intervention score for the use of feedback plans was noted to be the lowest of the survey items, which contributed to the dramatic increase in score. Residents are likely less familiar with feedback action plans compared to SMART goals, the application and utility of feedback, and other facets of feedback, which contributed to the low pre-intervention score. These outcomes align with self-determination theory, particularly in the domains of competence and independence, which are necessary for motivation and the formation of a professional identity [18]. By giving residents a framework to initiate feedback, the session fostered a sense of ownership over their learning and encouraged deeper engagement with clinical education.

Despite these gains, some outcomes revealed persistent challenges. Residents continued to report emotional discomfort with feedback, particularly the tendency to take it personally. This response reflects the complex interplay between feedback and self-concept, where perceived criticism can trigger ego defense mechanisms [19]. The persistent emotional discomfort reported by residents in our study aligns with broader observations that learners vary widely in how they engage with and process educational experiences, underscoring the importance of designing curricula that address not only cognitive but also affective dimensions of learning [20]. Additionally, PGY-3 residents demonstrated lower retention of workshop content, possibly due to the timing or perceived irrelevance of the material at their stage of training. These findings suggest the need for longitudinal reinforcement of feedback skills and tailored interventions that address emotional resilience and timing of delivery. Future iterations may benefit from integrating reflective exercises and peer coaching to normalize feedback as a growth tool [21].

EM programs should consider implementing structured feedback literacy workshops early in residency, ideally during intern orientation. Emphasizing learner‑driven strategies, such as clarifying expectations, setting SMART goals, and scheduling feedback check‑ins, can foster a culture of proactive engagement and continuous improvement. Integrating these workshops with broader adaptive, learner‑centered educational approaches may further strengthen residents’ ability to engage deeply and confidently in the feedback process [20]. Programs may also benefit from revisiting these concepts annually to reinforce retention and adapt to evolving learner needs. Addressing the emotional complexity of feedback through faculty development and peer support can further enhance its effectiveness. Ultimately, empowering residents to take an active role in the feedback process supports their development into reflective, competent, and patient‑centered physicians.

This study has several limitations. Its single‑site design and small sample size may limit generalizability, though the results offer valuable early insights. Because retention testing and survey participation occurred immediately after the workshop, the freshness of the material may have contributed to higher scores. The study focused on short‑term outcomes, so long‑term retention and sustained behavioral or clinical impact remain areas for future investigation. Additionally, the absence of a control group limits direct comparison; however, the pre‑post design still provides meaningful evidence of improvement associated with the intervention.

## Conclusions

This study suggests that a structured, learner-centered workshop can improve EM residents’ self-reported comfort and confidence in seeking and receiving feedback, as well as their use of practical strategies such as clarifying expectations, setting SMART goals, and creating actionable feedback plans. By emphasizing learner-driven engagement rather than faculty-only delivery, this intervention addresses a gap identified in the literature and offers a replicable framework for promoting feedback literacy early in training. Future research should investigate the durability of these skills, the effect of repeated exposure, and how these strategies are applied in clinical practice. By fostering feedback literacy early in training, residency programs can cultivate reflective, resilient, and growth-oriented physicians.

## Appendices

Appendix 1

Receiving Feedback Pre- and Post Comfort Survey

Please answer the following questions on a scale of 1-5 (Strongly Disagree to Strongly Agree).

I know how to clarify expectations at the start of a new rotation.

I know how to create SMART goals.

I take feedback personally.

I am comfortable receiving constructive feedback.

I actively seek out feedback.

I know how to create a feedback action plan at the start of each rotation.

I reflect upon and implement feedback.

Feedback is important for my growth and development.

Receiving and implementing feedback regarding my clinical performance improves patient care.

Obtaining and implementing feedback is the learner's responsibility.

Appendix 2

Receiving Feedback Knowledge Assessment

Expectations

What is the primary purpose of receiving feedback in medical education?

A.      To identify areas for growth, areas of strength, and improve patient care (Correct Answer)

B.      To fulfill program requirements

C.     To remediate residents

D.     To compare residents

When should residents ideally ask questions to clarify expectations?

A.      At the end of the rotation

B.      Only during evaluations

C.     At the beginning of each shift or rotation (Correct Answer)

D.     After receiving feedback

What is the role of the resident in the receiving feedback process?

A.      Passive recipient

B.      Active participant (Correct Answer)

C.     Evaluator

D.     Observer

Goal Setting

What does the S in SMART goals stand for?

A.      Sustainable

B.      Specific (Correct Answer)

C.     Strategic

D.     Simple

What are the benefits of SMART Goals?

A.      Encourages self-reflection and identification of knowledge gaps

B.      Promotes actionable feedback from supervisors

C.     Improves overall growth of learners

D.     Improves patient care and outcomes

E.      All the above (Correct Answer)

What is a recommended practice when setting goals with faculty?

A.      Wait until the end of the rotation to discuss goals

B.      Create goals independently

C.     Collaborate at the start of the shift to set goals (Correct Answer)

D.     Avoid goal setting

Feedback Action Plan

What is the benefit of a mid-point check-in during a shift or rotation?

A.      To finalize evaluations

B.      To adjust goals and receive feedback, ultimately improving patient care (Correct Answer)

C.     To reduce workload

D.     To avoid final feedback

What are the benefits of a feedback action plan?

A.      Demonstrates engagement and growth mindset

B.      Sets clear expectations for both the learner and educator

C.     Creates a plan to hold both parties accountable

D.     Improves quality and quantity of feedback

E.      All the above (Correct Answer)

You are starting a 4-week rotation, and you want to improve your skills in this specialty. You will be working with the same fellow for the entire month. When is the ideal time to discuss your performance and feedback?

A.      Timing discussed in Feedback Action Plan (Correct Answer)

B.      First Day

C.     End of Rotation

D.     To One Week
